# Lecithin-based wet chemical precipitation of hydroxyapatite nanoparticles

**DOI:** 10.1007/s00396-015-3557-0

**Published:** 2015-03-15

**Authors:** Wojasiński Michał, Duszyńska Ewa, Ciach Tomasz

**Affiliations:** BioMedical Engineering Laboratory, Faculty of Chemical and Process Engineering, Warsaw University of Technology, Waryńskiego 1, 00-645 Warszawa, Poland

**Keywords:** Hydroxyapatite, Nanoparticles, Synthesis, Lecithin, Wet chemical precipitation

## Abstract

Hydroxyapatite Ca_10_(PO_4_)_6_(OH)_2_ nanoparticles have been successfully synthesized by the wet chemical precipitation method at 60 °C in the presence of biocompatible natural surfactant—lecithin. The composition and morphology of nanoparticles of hydroxyapatite synthesized with lecithin (nHAp-PC) was studied by X-ray diffraction (XRD), Fourier transform infrared spectroscopy (FTIR), and scanning electron microscopy (SEM). Size distribution for nanoparticles was measured by nanoparticle tracking analysis in NanoSight system. We discuss in details influence of lecithin concentration in reaction system on nHAp-PC morphology, as well as on size distributions and suspendability of nanoparticles. Product exhibits crystalline structure and chemical composition of hydroxyapatite, with visible traces of lecithin. Difference in surfactant amounts results in changes in particles morphology and their average size.

## Introduction

In current research, the calcium phosphate materials, including hydroxyapatite (HAp), gather a lot of attention. Biocompatibility, osteoinductivity, osteoconductivity, and non-toxic properties of HAp create an opportunity to apply this material in wide range of industries [1]. Beginning with biomedical applications, HAp in different physical forms finds application in tissue engineering [2], drug delivery [3], implants [4], and bone cements [5]. High stability of HAp in a wide range of physical conditions makes it a desirable material for other industries. Apart from biomedical application, one can utilize HAp in catalysis [6], in adsorption processes [7], or in chromatography [8]. For the most cases of its applications, hydroxyapatite has a form of nanoparticles (nHAp), sometimes with different sizes and morphologies: from plates, through rod-shape nanoparticles, to spherical nanoparticles.

In order to apply any kind of material for practical usage, there is a great need to produce it quickly, without high-cost manufacturing equipment and in stable, controllable conditions. In the case of nHAp synthesis, the methods might be divided into three categories: (1) simple chemical processes, (2) advanced processes, and (3) processes with application of novel technologies. Such a division depends on the complication of the synthesis system or difficulties on maintaining the synthesis conditions. Looking at the simplest approaches for nHAp synthesis—mostly one-step processes—one can extract as the most common wet chemical precipitation technique [9–11], hydrothermal method [12, 13], sol-gel method [14], and microemulsion method [15]. In all of these methods, addition of templates, surfactants, or crystallization mediation agents is possible for improvement of product physical properties control. However, for applications in field of biomedical engineering, nHAp need to be synthesized without addition of any toxic additives. Biomimicking process [16], aerosol pyrolysis [17], and combustion preparation [18] are the examples of the advanced processes. Effort made for those advances aimed on higher nHAp quality in terms of morphology, connected with its application. Nevertheless, the cost of nHAp synthesis in this manner has risen. The last group of nHAp synthesis processes contains methods using novel energy sources for nanoparticle formation: ultrasounds [19], microwaves [20], and laser ablation [21]. Those methods yield with the highest quality of the nHAp (narrow size distribution, uniform particles morphology, etc.), on the other hand, rise the production cost.

Product properties, chemical as well as physical, play a key role in its application. Synthesis process of the product should give a possibility to control product properties in a reproducible and dependable manner, without cost rising. Among simple chemical processes, wet chemical precipitation method for synthesis of hydroxyapatite nanoparticles has raised up much interest owing to its good repeatability, low reaction temperature, and reaction system simplicity [9]. In wet chemical precipitation of nHAp, chemical and morphological properties of the product can be tailored by variation of synthesis conditions: temperature; pressure; and pH [22], as well as by additives, polymers, surfactants, and small molecules. Research groups tested various polymers in order to control morphology of the synthesized hydroxyapatite nanoparticles or to provide a template for synthesis, including the following: polyvinyl alcohol (PVA) [16], polyethylene glycol (PEG) [9], polystyrene-acetoacetoxyethyl methacrylate (PS-AAEM) [1], etc. Addition of surfactants into synthesis system is another way of product properties control and includes substances like the following: cetyltrimethylammonium bromide (CTAB) [9], sodium dodecyl sulfate (SDS) [10], and ethanolamine [22]. In some cases of wet chemical precipitation process and hydrothermal method, one can add small molecule substances like sodium citrate to affect physical characteristics of nHAp [13].

In light of currently used technologies described above, we propose a novel approach to the wet chemical precipitation method for synthesis of hydroxyapatite nanoparticles. The goal of this paper is to describe the wet chemical precipitation process based on the application of solution of lecithin—zwitterionic surfactant, mixture of phosphatidylcholines (PC) ubiquitous in cellular membranes—as a controlling agent of product properties. Proposed process results with hydroxyapatite nanoparticles chemically modified with lecithin—a product suitable for various applications. Furthermore, phosphatidylcholines bonded to the nanoparticle surface increase biocompatibility and suspendability of the synthesis product. Additionally, the paper presents results of investigation of the product chemical and crystalline structure (by Fourier transform infrared spectroscopy and X-ray diffraction) and morphology (by scanning electron microscopy) as a function of the amount of lecithin added to the precipitation system. We present size distributions and results of the estimation of suspendability property of the resulting nanoparticle powders in water (estimated by NanoSight measurements). Method for production of nanoparticles of hydroxyapatite bonded with lecithin (nHAp-PC) presented in this paper yields with product for many kinds of applications: from biomedical products to chemicals from and for various industries.

## Materials and methods

### Materials

Diammonium phosphate (NH_4_)_2_HPO_4_, calcium nitrate tetrahydrate Ca(NO_3_)_2_·4H_2_O, ammonium water NH_3_∙H_2_O (Chempur, Poland), lecithin (Serva, Germany), and commercial hydroxyapatite nanopowder (particle size <200 nm, Sigma-Aldrich, Germany) were used as received without any further purification.

### Synthesis of HAp-PC nanoparticles

nHAp-PC nanoparticles were synthesized by the wet chemical precipitation method, under atmospheric pressure, with calcium nitrate (Ca(NO_3_)_2_·4H_2_O) as calcium source and diammonium phosphate ((NH_4_)_2_HPO_4_) as phosphorous source and lecithin as an additive to control product properties. (NH_4_)_2_HPO_4_, Ca(NO_3_)_2_·4H_2_O were dissolved in demineralized water, while lecithin was suspended. In synthesis process, the Ca/P molar ratio was maintained at 1.67, corresponding to molar ratio in natural-sourced hydroxyapatite, and pH value of reaction mixture was adjusted to 10.0 with aqueous ammonia NH_3_∙H_2_O. Level of pH in reaction system promotes nHAp nucleation through the following mechanism [16]:

$$ 10{\mathrm{Ca}}^{2+}+6{\mathrm{PO}}_4^{3-}+2{\mathrm{OH}}^{-}\to {\mathrm{Ca}}_{10}{\left({\mathrm{PO}}_4\right)}_6{\left(\mathrm{O}\mathrm{H}\right)}_2 $$

Reaction procedure was as follows: 0.0075 moles of Ca(NO_3_)_2_·4H_2_O was dissolved in 25 ml of demineralized water. Suspensions of lecithin in 37.5 ml of demineralized water were prepared, with concentrations 0.30, 0.75, 1.50, 3.00, and 9.00 % *w/w*. Ca(NO_3_)_2_·4H_2_O solution was added into reactor, and lecithin suspension was slowly and gently added to the reaction system. The initial value of pH of the resulting solution was adjusted to 10.0 with ammonia water. The temperature was controlled and set at 60 °C and mixture was stirred to maintain stable conditions. Then, 0.0046 moles of (NH_4_)_2_HPO_4_ was dissolved in 15 ml of demineralized water and the initial pH of the solution was adjusted to 10.0. Diammonium phosphate solution was added drop-wise into the mixture of calcium nitrate and lecithin at a feeding rate of 2 ml/min during the stirring. Then, mixture was stirred continually for 1.5 h in 60 °C, and then naturally cooled to room temperature and left under stirring overnight for aging the product. The resulting suspension was centrifuged (Centrifuge MPW-251, MPW Med.Instruments, Poland), decantated, and washed with hexane (washing process was repeated five times) to remove unbounded residues of lecithin. The final product (yellowish powder) was dried at 50 °C for 24 h.

### Measurements

The values of initial pH were measured with SevenMulti (Mettler Toledo, Switzerland) with measuring range of pH 0.000–14.000, resolution 0.001, and accuracy ±0.002. The degree of crystallinity was characterized by X-ray diffraction (XRD, D8 Advanced, Bruker-ASX) using Cu Kα radiation (*λ* = 0.15406 nm) at the X-ray tube voltage 40 kV and tube current 40 mA. The XRD data were collected at room temperature over the 2θ range of 3–60° at a step size of 0.02°/s. The characteristic groups of HA and lecithin were analyzed by Fourier transform infrared spectroscopy (FTIR, NicoletTM 6700 spectroscope, Thermo Scientific, USA). Morphology of final dry product was studied by scanning electron microscope (SEM, Zeiss Ultra Plus, Germany). Average size, size distribution, and assessment of suspendability of synthesized nHAp-PC powders were carried out based on nanoparticle tracking analysis (NTA). For the analysis, NTA 2.3 software on NanoSight system (LM10, Malvern Instruments Ltd., UK) was applied. The system measures diffusion coefficient for particles and then calculates, from Stokes-Einstein equation, their sphere-equivalent hydrodynamic diameter. In this paper, calculated hydrodynamic diameter is considered as a size of the analyzed nanoparticles. Powder samples were suspended, by sonication for 10 min, in ultra pure water (Direct-Q System, Merck Millipore, Germany) to obtain 10 ml of highly diluted suspension prior NTA measurements.

## Results and discussion

**Fig. 1:**
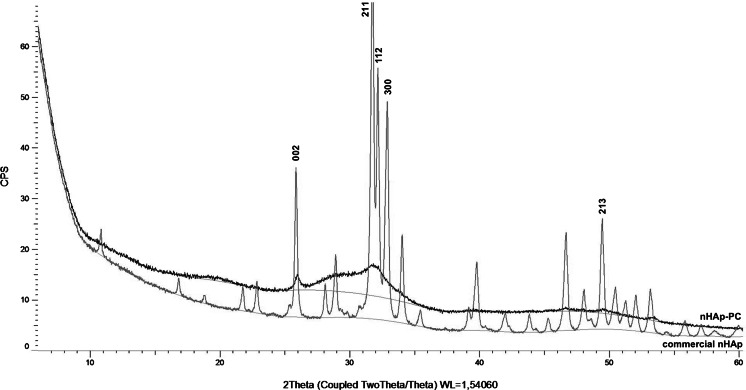
XRD patterns of commercially available hydroxyapatite powder (nHAp) and hydroxyapatite nanoparticles (nHAp-PC) synthesized with 3.00 % *w*/*w* lecithin

The XRD diffraction pattern of nHAp-PC synthesized in the presence of lecithin and diffraction pattern of commercially available nHAp are shown in Fig. 1. Comparison with XRD pattern of nHAp and data in standard JCPDS file no. 09-0432 obtained for pure crystalline hydroxyapatite proved consistency between two samples. Some differences in peak intensity and positions between analyzed nHAp-PC sample and commercial nHAp, as well as literature reference, might be caused by: (1) amorphous structure, (2) preferred orientation of small crystallites in studied powder; (3) crystallites shape anisotropy—different size in different crystalline directions; (4) crystallites microstress anisotropy—atomic systematic displacement in lattice node; and (5) presence of lecithin bonded to surface of the nHAp-PC. Despite the fact of those slight differences in the crystallinity of nHAp-PC comparing to commercial nHAp, and HAp form data file, the structure of nHAp-PC corresponds with previously reported hydroxyapatite nanoparticles obtained in different manners [9, 12, 20].

**Fig. 2:**
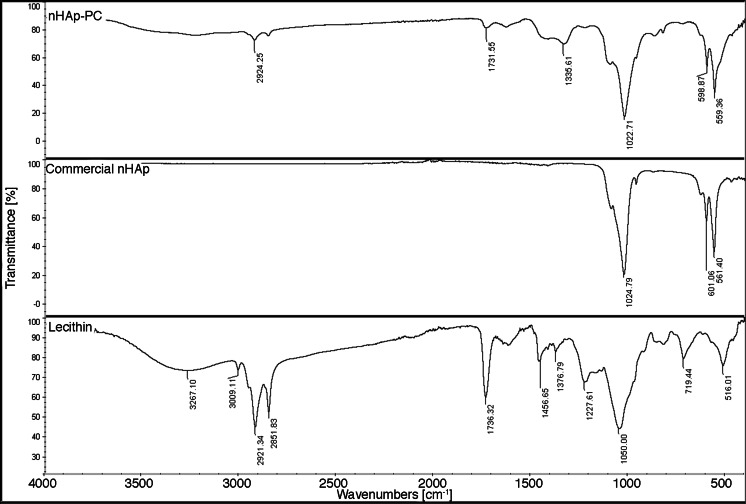
FTIR spectra of lecithin-based (3.00 % *w*/*w*) wet chemical precipitation synthesized hydroxyapatite nanoparticles (nHAp-PC), commercially sourced hydroxyapatite nanoparticles (nHAp), and pure soybean lecithin

FTIR analysis of nHAp-PC shows peaks position consistent with commercially available hydroxyapatite (Fig. 2). The strongest, characteristic peak for nHAp derived from PO_4_^3−^ appears at position of 1,030 cm^−1^ for commercially available nHAp and nHAp-PC produced in lecithin-based wet chemical precipitation. Peaks derived from P-O stretching bonds appear at 570 and 609 cm^−1^ for nHAp and nHAp-PC, which indicate correspondence and chemical similarity of commercially available hydroxyapatite and hydroxyapatite synthesized in the presence of lecithin. Spectra of lecithin shows peak from C = O stretching bonds in ester groups at 1,737 cm^−1^, peaks from C-O stretching bond at 1,376 and 1,227 cm^−1^, and peaks from C-H bond of fatty-acid’s long chains at 2,852 and 2,921 cm^−1^ (Fig. 2). The weak bands corresponding to lecithin appear in nHAp-PC spectra, which suggest presence of lecithin bonded to the surface of the final product. The effect of surfactant bonding to the resulting nanoparticles is established in literature. Liu et al. [9] reported presence of residues of cetyltrimethylammonium bromide (CTAB)—toxic surfactant—in hydroxyapatite nanoparticles synthesized in wet chemical precipitation. Apart from lecithin presence, the results of chemical composition analysis of nHAp-PC are comparable with results obtained for other methods for synthesis of nanoparticles of hydroxyapatite [20, 23]. Biocompatibility of lecithin is well established in the literature [24, 25]; thus, its presence in the synthesized nHAp-PC might be preferable for its biomedical applications.

**Fig. 3:**
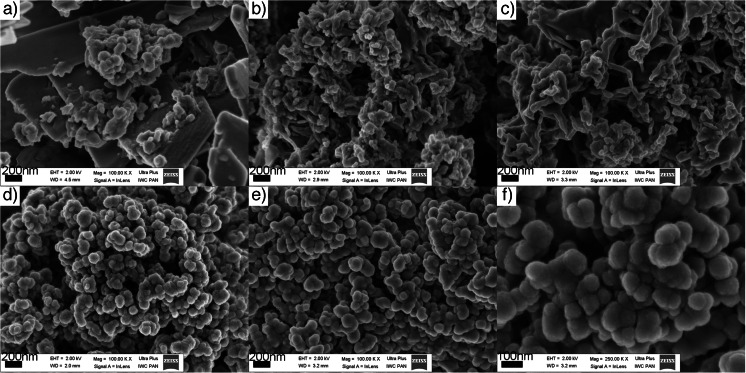
SEM images of lecithin-based wet chemical precipitation synthesized hydroxyapatite nanoparticles: **a** 0.30 % *w*/*w* lecithin, **b** 0.75 % *w*/*w* lecithin, **c** 1.50 % *w*/*w* lecithin, **d** 3.00 % *w*/*w* lecithin, **e** 9.00 % *w*/*w* lecithin, and **f** enlargement of 9.00 % *w*/*w* lecithin; note the difference in scale

Scanning electron microscope images in Fig. 3 present the morphology of nHAp-PC particles synthesized in the system with amount of lecithin in the range from 0.30 to 9.00 % *w*/*w*. We observed dependence of nHAp-PC particles morphology as a function of amount of lecithin added to synthesis system in proposed process. Part (a) in Fig. 3 shows the plate-like morphology, mostly nanoplates with thickness up to hundreds of nanometers, as a dominant form. But, we noticed some accompanying particles without specific geometrical form. The nanoplate morphology of the nHAp-PC corresponds with the lowest, in investigated range, amount of added lecithin—0.30 % *w*/*w*. With increasing the lecithin concentration through 0.75 to 1.50 % *w*/*w*, we obtained nanoparticles in form of nanorods. In part (b) of Fig. 3, we present how for increasing amount of lecithin, nanoparticles, become thinner in diameter and longer. Further increase of lecithin amount in the synthesis system, to 3.00 and 9.00 % *w*/*w*, resulted with particles in form of nanospheres. Based on SEM images (Fig. 3d–f), we noticed two effects for these powders: (1) increased lecithin concentration to 9.00 % *w*/*w* caused formation of bigger nanoparticles than for 3.00 % *w*/*w*; (2) the adhesion-like effect appears between the nHAp-PC, particles form cauliflower-like structure. The second observation inspired us to investigate the nanoparticles suspendability in aqueous suspensions.

**Fig. 4:**
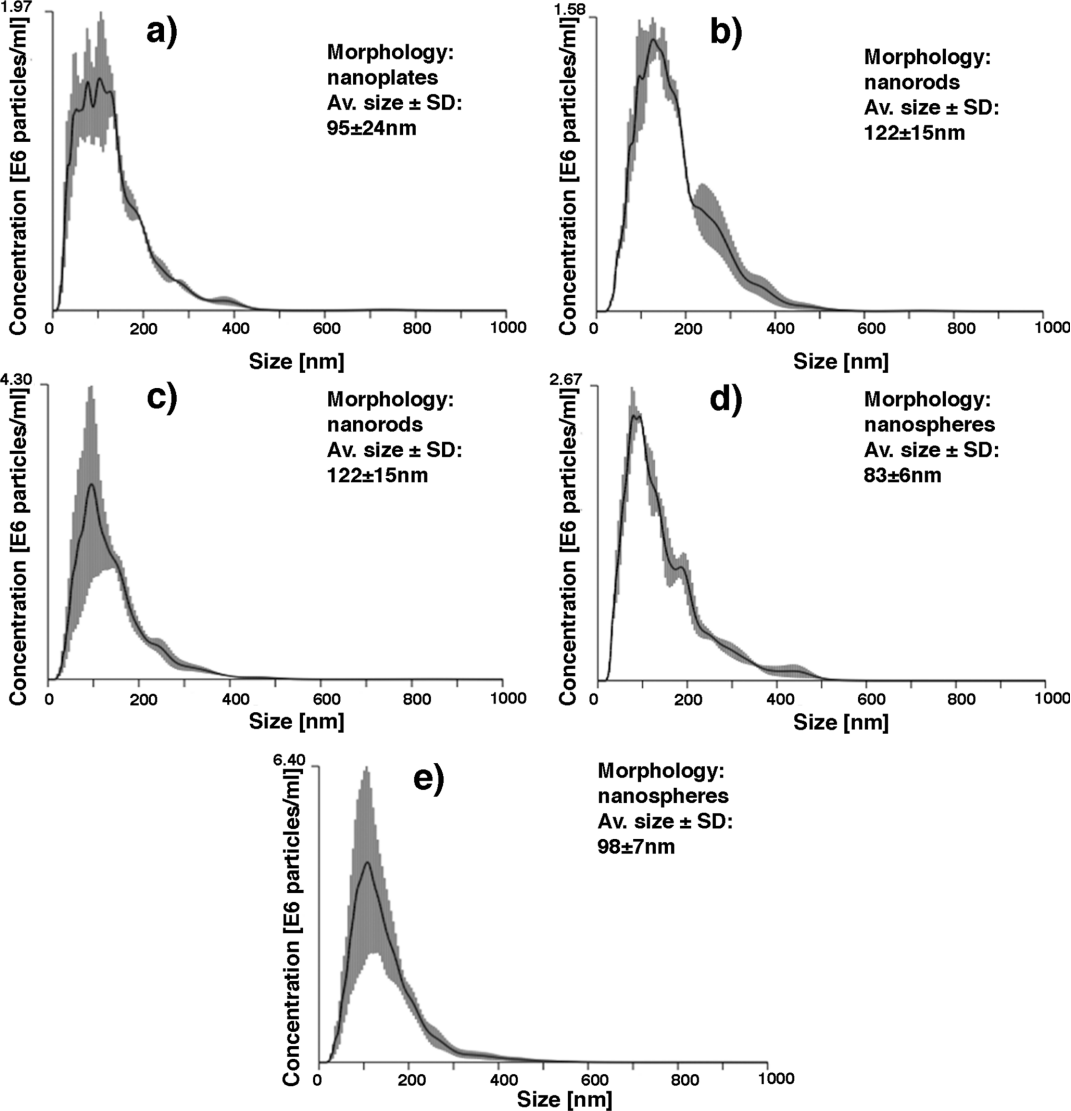
NanoSight size distribution, dominant particle morphology, and average particle size of lecithin-based wet chemical precipitation synthesized hydroxyapatite nanoparticles: **a** 0.30 % *w*/*w* lecithin, **b** 0.75 % *w*/*w* lecithin, **c** 1.50 % *w*/*w* lecithin, **d** 3.00 % *w*/*w* lecithin, **e** 9.00 % *w*/*w* lecithin

Using nanoparticles tracking analysis in NanoSight system, we recorded size distributions for all synthesized nHAp-PC (Fig. 4). Since the particles synthesized in the system with lecithin amount 0.30, 0.75, and 1.50 % *w*/*w* are non-spherical in shape, the results of average size measurement (particles were treated by the system as sphere-equivalent in morphology) are only the estimation for these particles appearance in aqueous environment. Based on SEM images, we estimated the average sizes of the nanoplates in height and width on about 1 to 4 μm, and the length of nanorods produced with addition of 0.75 and 1.50 % *w*/*w* of lecithin on about 100 to 400 nm and about 200 to 1 μm, respectively. Comparing the results of SEM measurements with NanoSight analysis, we were able to confirm presence of small, heterogeneous in shape particles in nanoplate samples, where the plates with nanosized thickness were not measured by NTA software (plates were over 1 μm in size—over the limit of the system). In the case of nanorod samples, for nHAp-PC synthesized with 0.75 % *w*/*w* addition of lecithin, we obtained the bimodal size distribution, which suggest presence of nanorods measured by system simultaneously in both directions. In all previously mentioned instances, the particles in suspension were well separated. For particles synthesized with 3.00 and 9.00 % *w*/*w* addition of lecithin—spherical nanoparticles, the average size we obtained is comparable with results from SEM measurements. SEM images analysis results with dimensions of nHAp-PC synthesized with 3.00 % *w*/*w* of lecithin about 40 to 150 nm and dimensions of nHAp-PC synthesized with 9.00 % *w*/*w* of lecithin about 60 to 250 nm. However, the average diameter of those particles measured in NanoSight system is about 75 to 90 and 90 to 110 nm, respectively. The size distributions recorded for nanospheres of hydroxyapatite obtained in presence of lecithin show the portion of the biggest particles about 400 nm. Since the aggregates visible in the SEM image (Fig. 3f) are much bigger (even 1 μm in diameter), we postulate that nHAp-PC might form proper suspension of nanoparticles in aqueous media.

The possibility to control product properties like morphology, size of particles, is a goal reported in many papers. In the case of the spherical nanoparticles—the most reported morphology of nHAp—the particles we obtain in lecithin-based process correspond with particles synthesized in processes controlled in other way. Wang et al. [22] reported hydroxyapatite nanoparticles formation in wet chemical precipitation with different solvents with particle average size up to 300 nm. Using different simple organic small molecules, Schiff bases, Mohandes et al. [26] obtained particles with sizes about 30 to 300 nm. On the other hand, Kramer et al. [11], just by simple control of reactants addition rate, synthesized spherical hydroxyapatite nanoparticles with average size about 30 to 250 nm. Comparing to other approaches to wet chemical precipitation technique of synthesis of hydroxyapatite nanoparticles, we propose simple system with application of nontoxic surfactant—lecithin—to control not only the size, but morphology too. For more detailed comparison of discussed techniques, please see Table 1. Cytotoxicity of surfactants like CTAB or sodium dodecyl sulfate (SDS) results from their interactions with cellular membranes leading to cell wall rupture. This effect might be observed even when surfactants are bonded or adsorbed on the biomaterial surface. Lecithin—mixture of phosphatidylcholines, components of cellular membranes [24]—when present on the surface of any biomaterial, attracts cells to interact with the material without causing any damage to the cell membrane [25]. Aforementioned reported approaches to wet chemical precipitation allow to affect the particles morphology, but only in range from nanorods to nanopheres. It is important to notice that morphology control—in specific way—in the nHAp formation processes requires more complicated solutions, like previously mentioned, e.g., microemulsions, microwaves or laser ablation [15, 20, 21].

**Table 1:** Selected methods of morphology and size control in nHAp synthesis reported in literature

Synthesis of nHAp	Morphology and size control method	Particles morphology	Particles size [nm]	Ref.
Wet chemical precipitation	Different solvents	Dominant rod; sphere	~300	[22]
Wet chemical precipitation	Addition of Schiff bases	Dominant sphere; rod	30–300	[26]
Wet chemical precipitation	Reactant addition rates (titration speed)	Shift from rod to sphere	30–250	[11]
Microemulsions	Emulsions system	Sphere-like	30–350	[15]
Microwaves	Application of microwaves	Sphere	up to 40 nm	[20]
Laser ablation	Gas-phase laser ablation	Sphere	up to 100	[21]

## Conclusions

In this paper, we proposed a novel approach to wet chemical precipitation of hydroxyapatite nanoparticles in the presence of lecithin in the reaction system. We showed resulting nanoparticles crystallinity and chemistry analysis results, proving that lecithin-based approach allows formation of good quality hydroxyapatite nanoparticles, slightly modified by traces of lecithin (nHAp-PC). Based on SEM image investigation and additional particle tracking analysis experiments in NanoSight system, we described possibilities to control the product morphology and the average particle size by varying lecithin concentration in the reaction system only. We postulate the increased biocompatibility of the resulting nHAp-PC material by modification of nanoparticles by bonding with lecithin. This modification also increases suspendability property of nanoparticles in various media.

The patent of the presented method is pending.
